# Exploring immediate cardiorespiratory responses: low-intensity blood flow restricted cycling vs. moderate-intensity traditional exercise in a randomized crossover trial

**DOI:** 10.1186/s13102-024-00951-0

**Published:** 2024-08-15

**Authors:** Manuel Kuhn, Christian F. Clarenbach, Adrian Kläy, Malcolm Kohler, Laura C. Mayer, Martin Lüchinger, Belinda Andrist, Thomas Radtke, Sarah R. Haile, Noriane A. Sievi, Dario Kohlbrenner

**Affiliations:** 1 Faculty of Medicine, University of Zurich, Zurich, Switzerland; 2 Department of Pulmonology, University Hospital Zurich, Raemistrasse 100, 8091 Zurich, Switzerland; 3 Epidemiology, Biostatistics and Prevention Institute, University of Zurich, Zurich, Switzerland

**Keywords:** Blood flow restriction training, Occlusion, Interval training, Cycling exercise, Cardiorespiratory response, Healthy individuals

## Abstract

**Purpose:**

Blood-flow restriction (BFR) endurance training may increase endurance performance and muscle strength similar to traditional endurance training while requiring a lower training intensity. We aimed to compare acute cardiorespiratory responses to low-intensity interval exercise under BFR with moderate-intensity traditional interval exercise (TRA).

**Methods:**

We conducted a randomized crossover study. The protocol involved three cycling intervals interspersed with 1 min resting periods. With a 48-h washout period, individuals performed the protocol twice in random order: once as BFR-50 (i.e., 50% incremental peak power output [IPPO] and 50% limb occlusion pressure [LOP]) and once as TRA-65 (65% IPPO without occlusion). TRA-65 intervals lasted 2 min, and time-matched BFR-50 lasted 2 min and 18 s. Respiratory parameters were collected by breath-by-breath analysis. The ratings of perceived breathing and leg exertion (RPE, 0 to 10) were assessed. Linear mixed models were used for analysis.

**Results:**

Out of the 28 participants initially enrolled in the study, 24 healthy individuals (18 males and 6 females) completed both measurements. Compared with TRA-65, BFR-50 elicited lower minute ventilation (VE, primary outcome) (-3.1 l/min [-4.4 to -1.7]), oxygen consumption (-0.22 l/min [-0.28 to -0.16]), carbon dioxide production (-0.25 l/min [-0.29 to -0.20]) and RPE breathing (-0.9 [-1.2 to -0.6]). RPE leg was significantly greater in the BFR-50 group (1.3 [1.0 to 1.7]).

**Conclusion:**

BFR endurance exercise at 50% IPPO and 50% LOP resulted in lower cardiorespiratory work and perceived breathing effort compared to TRA at 65% IPPO. BFR-50 could be an attractive alternative for TRA-65, eliciting less respiratory work and perceived breathing effort while augmenting perceived leg muscle effort.

**Trial registration:**

NCT05163600; December 20, 2021.

**Supplementary Information:**

The online version contains supplementary material available at 10.1186/s13102-024-00951-0.

## Introduction

Compared with traditional endurance training, blood-flow restriction (BFR) endurance training has emerged as a promising approach for enhancing endurance performance and muscle strength while utilizing substantially lower training intensities [1–4]. BFR is typically applied by a pneumatic cuff at the most proximal location of the target muscles. During BFR endurance exercise, the inflated cuff decreases venous outflow to the exercising limb, which causes an increase in metabolites and fluid within the limb [5, 6].

Low-intensity BFR endurance exercise leads to intramuscular/intracellular, cardiac and vascular adaptations [3, 6–8], which can enhance maximum oxygen uptake (VO_2max_) and improve endurance performance. BFR related training effects have been linked to various underlying mechanisms such as an increase in endothelium-dependent vasodilation, capillary density, vascular endothelial growth factor, AMPK signaling and a reduction of K + release from the contracting muscles [8–10].

Overall, longitudinal studies have shown that low-intensity BFR endurance training can produce effects on endurance performance comparable to those of high-intensity traditional endurance training ([3, 11–13].

In rehabilitative and recreational exercise settings, moderate intensity endurance training, typically at 65% of the incremental peak power output (IPPO), is generally favored because patients in rehabilitation often cannot sustain high-intensity exercise [14]. Therefore, especially for rehabilitation, lowering intensities by using BFR as a training method is very attractive, as this could lead to decreased mechanical stress and potentially lowered cardiorespiratory work. Recent studies have focused mainly on the comparison of methods that use either identical (i.e., low-intensity with BFR vs. low-intensity without BFR) or contrasting intensities (i.e., low-intensity with BFR vs. high-intensity without BFR).

Research in healthy individuals demonstrated that adding BFR to a low-intensity endurance exercise (i.e., ≤ 50% VO_2max_) leads to an increase in acute cardiorespiratory demands and ratings of perceived exertion (RPE) compared to the same exercise without BFR [15–17]. Additionally, comparisons between low-intensity BFR and high-intensity traditional endurance exercise (i.e., ≥ 80% VO_2max_) have shown that oxygen consumption (VO2), minute ventilation (VE), heart rate (HR), and muscle oxygenation are lower in low-intensity BFR, while RPE remains comparable [15, 17–20].

However, there has been no investigation into acute cardiorespiratory responses to low-intensity BFR exercise compared to moderate-intensity traditional exercise, which is commonly used in rehabilitation settings. Understanding this comparison is crucial for determining whether BFR can be effectively integrated into cardiopulmonary rehabilitation programs.

Hence, the objective of this study was to investigate differences in acute cardiorespiratory and perceptual responses between low-intensity BFR endurance exercise (BFR-50) and moderate-intensity traditional endurance exercise (TRA-65).

## Materials and methods

### Individuals

Individuals were recruited at the University Hospital Zurich, Switzerland, between January 2022 and September 2022 and had to be at least 18 years old and healthy. We excluded individuals who experienced pain during exercise of any origin, a history of thromboembolic events in the lower extremities, or a resting systolic blood pressure (BP) ≤ 100 mmHg or ≥ 140 mmHg. Furthermore, pregnant individuals and individuals with a mental or physical disability that precluded informed consent or compliance with the study protocol were excluded. The study was conducted in accordance with the Declaration of Helsinki, and all subjects provided written informed consent. The Ethics Committee of the Canton of Zurich approved the study (2021–02038). The study is registered on clinicaltrials.gov (NCT05163600). This study adheres to CONSORT guidelines [21].

### Experimental design

This was a single-center randomized crossover study (AB/BA) in which the main outcome was minute ventilation (VE, l/min). We selected VE as our primary outcome since it is a crucial factor that influences dyspnea in individuals with lung disease during exercise. Single exercise bouts cause transient perturbations in physiological homeostasis. However, they do not lead to sustained adaptations in human physiology and are therefore suitable for crossover trials. The endurance exercise stimuli we administered were of moderate or light intensity. Therefore, light-to-moderate degradation during intramuscular glycogen storage was assumed to occur, and glycogen would be recovered in less than 24 h [22]. In addition to these physiological considerations, we considered that individuals may feel a certain level of muscle soreness because they might not be familiar with the exercise modalities. To guarantee full recovery of physiological and subjective marker levels between the study visits, we administered a washout phase of ≥ 48 h. Further, participants were instructed to refrain from strenuous physical activity for 48 h prior to each exercise session to ensure a consistent physiological state. They were also asked to maintain their regular daily routines, including diet and hydration practices, and to get a consistent amount of sleep. To further control for circadian variations, all measurements were conducted at the same time of day for each participant.

The selection of a 50% IPPO intensity of BFR endurance exercise with a continuous occlusion pressure of 50% LOP during endurance exercise is based on the recommendation put forth by Patterson and colleagues [4]. For TRA-65, we selected a moderate intensity level of 65% IPPO, as recommended by the American College of Sports Medicine [14]. Individuals visited the laboratory on three separate occasions. Each visit lasted one hour.

*Visit 1* contained a screening procedure to verify whether the individuals were eligible for the study and to provide written informed consent. Eligible individuals underwent cardiopulmonary exercise testing (CPET) to assess VO_2peak_ and IPPO. Furthermore, individuals were randomized to the sequence BFR-50/TRA-65 or TRA-65/BFR-50. Height and weight were measured. The study setting was individually adapted to ensure that equipment and face masks fit properly (e.g., saddle height, handlebar reach, etc.). The settings were recorded to assure equal conditions on subsequent visits.

*Visits 2 and 3* were exercise visits. Individuals allocated to the TRA-65/BFR-50 sequence performed traditional interval cycling exercise without BFR at Visit 2 and interval cycling exercise with continuous BFR on both legs at Visit 3.

**Fig. 1:**
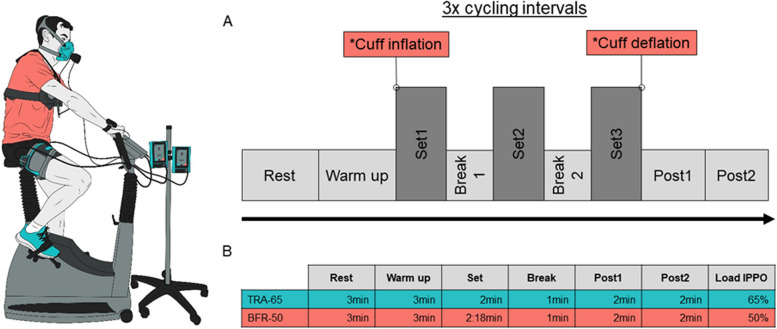
Overview of the intermittent cycling protocol (**A**) with a detailed description of traditional endurance exercise (TRA-65) and blood-flow restriction endurance exercise (BFR-50) (**B**). IPPO: Incremental peak power output; * Cuff inflation/deflation (only during BFR-50)

Individuals allocated to the sequence BFR-50/TRA-65 performed interval cycling exercise with continuous BFR on both legs at Visit 2 and traditional interval cycling exercise without BFR at Visit 3. Figure 1 shows a graphical representation of the study design.

### Cardiopulmonary exercise testing (Visit 1)

Upon arrival at the laboratory, the exact test protocol was explained to the participant. Volume and gas calibrations were performed before each test. CPET was performed in accordance with published guidelines [23]. Respiratory parameters (VE, VO_2_, carbon dioxide output [VCO_2_], tidal volume [VT], breathing rate [BR]) were collected breath-by-breath using a metabolic cart (Metamax 3b, Cortex Biophysik GmbH, Leipzig, Germany) [24]. In addition, continuous peripheral oxygen saturation (SpO_2_) with pulse oximetry (Wrist Ox2 3150, Nonin Medical, Minnesota, USA) [25] and heart rate (HR in beats per min [bpm]) with a chest belt (H10, Polar Electro OY, Kempele, Finland) [26] were recorded during CPET. Before and after the CPET, BP and RPE leg and breathing (numeric rating scale ranging from 0–10 with 0 representing “no leg fatigue” and “no shortness of breath” and 10 representing “maximum leg fatigue” and “maximum shortness of breath”, respectively were measured [27].

Initially, individuals rested for 3 min on a cycle ergometer (ergoselect 200, ergoline GmbH, Bitz, Germany) for collection of resting respiratory gas exchange and HR data, followed by a 3 min warm-up period of unloaded pedaling at 60 revolutions per minute (rpm). Subsequently, an incremental ramp exercise test was performed until exhaustion. The baseline load (25 to 75 W) and the increments in load per min (20 to 30 W) were individualized, depending on the self-reported training status of each individual and aiming for a test duration (i.e., the incremental ramp phase) of 8 to 12 min. The oxygen volume obtained immediately before the end of the incremental ramp exercise test was considered the VO_2peak_. IPPO was defined as the power output in watts at which VO_2peak_ was achieved during CPET. The IPPO was used to determine the exercise intensities at Visits 2 and 3.

### Main exercise trial (Visits 2 and 3)

Prior to the intermittent cycling protocol, individuals rested on the cycle ergometer for 3 min for the collection of baseline values. Following this baseline period, the individuals cycled for 3 min at 30% IPPO with ≥ 60 rpm (warm up). The pedaling frequency had to be kept constant during the sets.

TRA-65 consisted of 3 sets of cycling at 65% IPPO and a postexercise phase, Post1 and Post2, with a duration of each 2 min. Individuals were given the choice to either rest or pedal slowly (< 20 rpm) during breaks, Post1 and Post2, with encouragement to maintain consistent behavior throughout each condition.

BFR-50 consisted of 3 sets of cycling at 50% IPPO and a postexercise phase, Post1 and Post2, with a duration of each 2 min. We time-matched the interval duration between the two cycling protocols according to the difference in workload, i.e., 15% longer time intervals for BFR-50. Accordingly, the time limit was 2 min 18 s with a 1 min break. Individuals were given the choice to either rest or pedal slowly (< 20 rpm) during breaks, Post1 and Post2, with encouragement to maintain consistent behavior throughout each condition. The limb occlusion pressure (LOP) was set to 50%. Prior to the exercise, BFR cuffs were applied to the most proximal part of both legs. The cuffs were inflated at the start of Set1 after the warm-up period and remained inflated until the end of Set3. For an overview of the cycling protocol, see Fig. 1.

### Measurements during exercise training

During exercise, respiratory gas exchange variables and minute ventilation were measured breath-by-breath using a metabolic cart. In addition, SpO_2_ and HR were continuously recorded during the exercise protocol. RPE leg and breathing were measured immediately after each set and again 2 min and 4 min after the last set (i.e., Post1 and Post2).

### Limb occlusion pressure (LOP)

The limb occlusion pressure (LOP) was individually determined using an automatic tourniquet system (PTS for BFR, Delfi Medical Innovations Inc., Vancouver, Canada) while participants were in a relaxed supine position. Inflatable cuffs (Easy Fit BFR 11.5 × 86 cm, Delfi Medical Innovations Inc., Vancouver, Canada) were positioned around the most proximal part of each thigh, and the LOP was measured separately for each limb. During BFR-50, the system automatically applied cuff pressures equivalent to 50% of the LOP, with continuous adaptation to each limb separately throughout the three sets and two breaks. At the end of Set3, the cuffs were deflated.

### Statistical analysis

The data are presented as median (25th, 75th percentile) unless stated otherwise. All the statistical analyses were performed using R-4.3.1 on Windows (R Core Team 2023, R Foundation for Statistical Computing, Vienna, Austria). Linear mixed models were used to compare the course of all respiratory parameters, HR, and RPE between TRA-65 and BFR-50 and over three sets and two breaks, adjusted for treatment and period, and with random intercepts for each participant. Within-patient differences between the two conditions were used. The variance–covariance structure for the random effects was unstructured, as no correlation between participants was assumed. Restricted maximum likelihood estimation was used to fit the linear mixed effects models. Respiratory parameters, SpO_2_, RPE, and HR data were averaged across all distinct phases: "Set1," "Set2," "Set3," "Break1," and "Break2"respectively.

To account for the dynamic nature of respiratory responses observed during submaximal exercise, where a steady state is not always reached until 2 min into the exercise, we conducted a supplementary sensitivity analysis. This analysis used linear mixed models adjusted for treatment and period and included a random intercept for each participant. It involved averaging respiratory parameters, SpO_2_, RPE, and HR data over the final 20 s of each respective phase.

To illustrate the dynamic nature of respiratory responses, we depicted in supplementary figures the kinetics of VE, VO2, and VCO2 by averaging them over 10-s intervals during the whole exercise.

We powered our study to detect a moderate effect (i.e., an effect size of 0.6) in VE between the two exercise training regimens. Setting the power to 80% and the significance level to 5% led to a sample size of 24 individuals. Dropouts were replaced by new individuals who were individually randomized. If a dropout occurred after completing one of the two conditions in the first cycle, the available measurement was included in the analysis. After Visit 1, eligible individuals were randomly assigned to their exercise sequence with computer-generated permuted block randomization with random block sizes of 2 to 4 using the blockrand package in R [28].

## Results

### Sample characteristics

**Fig. 2:**
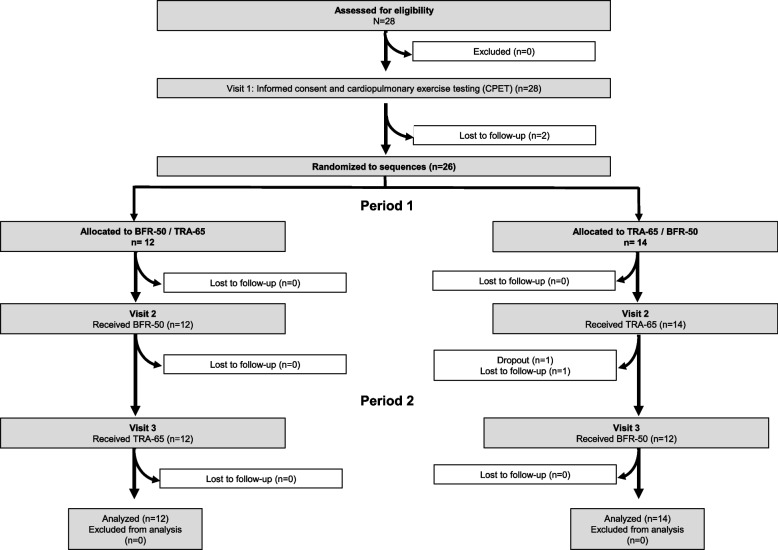
Flow chart: Three individuals could not be contacted anymore, and one individual had a hamstring injury that was not associated with the study. BFR-50: Blood-flow restriction endurance exercise; TRA-65: Traditional endurance exercise

A total of 28 individuals (8 females, 18 males) were included in the study. Of these, three individuals were lost to follow-up, and one individual withdrew consent due to a hamstring injury, which was not related to the study. Thus, 24 individuals completed all the examinations, and two individuals completed only TRA-65. Overall, 26 individuals were included in the analysis (Fig. 2). The characteristics of the overall study population stratified by test sequence are given in Table 1. No adverse event associated with this study occurred.

**Table 1:** Sample characteristics

	**BFR-50/TRA-65 (*n* = 12)**	**TRA-65/BFR-50 (*n* = 14)**	**Overall (*n* = 26)**
**Sex**			
Male	7 (58.3%)	11 (78.6%)	18 (69.2%)
Female	5 (41.7%)	3 (21.4%)	8 (30.8%)
**Age (years)**	30.5 [29.0, 34.5]	29.5 [25.0, 32.0]	30.0 [28.0, 33.3]
**BMI (kg/m**^**2**^**)**	22.6 [21.4, 23.9]	23.2 [22.3, 24.3]	23.1 [22.0, 24.2]
**LOP (mmHg)**			
Left leg	166 [160, 186]	180 [166, 206]	168 [164, 202]
Right leg	159 [153, 180]	182 [164, 200]	164 [158, 198]
**IPPO (Watt)**	309 [241, 336]	248 [219, 295]	264 [219, 322]
**VE**_**peak**_** (l/min)**	130 [109, 149]	128 [108, 154]	129 [108, 154]
**VO**_**2peak**_** (ml/min/kg)**	51.5 [43.8, 53.3]	45.0 [37.5, 49.3]	47.0 [39.8, 52.0]
**VO**_**2peak**_** (l/min)**	3.70 [2.91, 3.99]	3.30 [2.71, 3.51]	3.34 [2.71, 3.90]
**VCO**_**2peak**_** (l/min)**	4.13 [3.19, 4.49]	3.53 [3.10, 3.99]	3.78 [3.10, 4.31]
**Heart rate**_**peak**_** (bpm)**	177 [157, 187]	184 [176, 188]	182 [176, 188]

Data are median (25th and 75th percentile) or n (%). BFR-50: low-intensity BFR endurance exercise, *TRA-65* Moderate-intensity traditional endurance exercise, *BMI* Body mass index, *LOP* Limb occlusion pressure, *IPPO* Incremental peak power output, *VO*_*2peak*_ Peak oxygen consumption, *VE*_*peak*_ Peak ventilation at VO_2peak_, *VCO*_*2peak*_ Peak carbon dioxide output at *VO*_*2peak*_, Heart rate_peak_ Peak heart rate (beats per minute) at VO_2peak_

### Acute cardiorespiratory and perceptual response

**Fig. 3:**
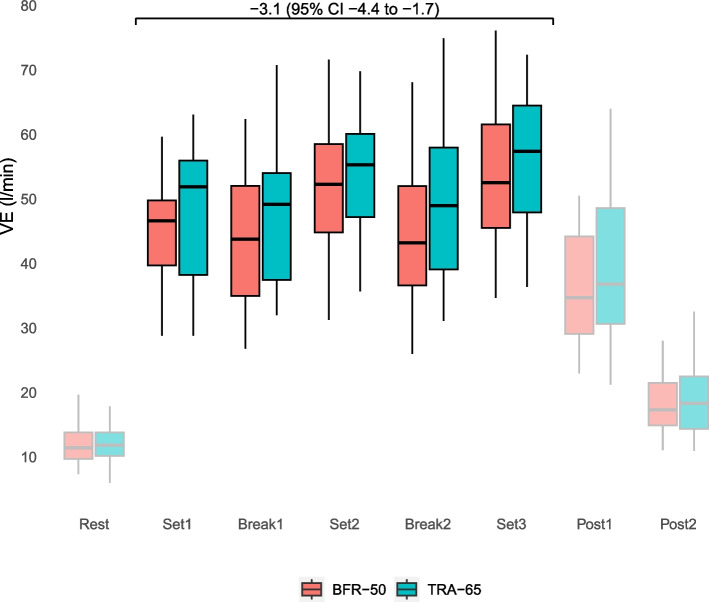
Time course of alterations in VE during intermittent cycling exercise as measured by BFR-50 (red) and TRA-65 (green). The annotation displays mean difference (95% CI) from linear mixed regression modeling. VE: minute ventilation; BFR-50: Blood-flow restriction endurance exercise; TRA-65: Traditional endurance exercise. The box plots display the median (line inside the box), interquartile range (IQR, edges of the box), and whiskers (lines extending from the box). Whiskers represent the range within 1.5 times the IQR from the 25th percentile (Q1—1.5IQR) to the 75th percentile (Q3 + 1.5IQR)

**Fig. 4:**
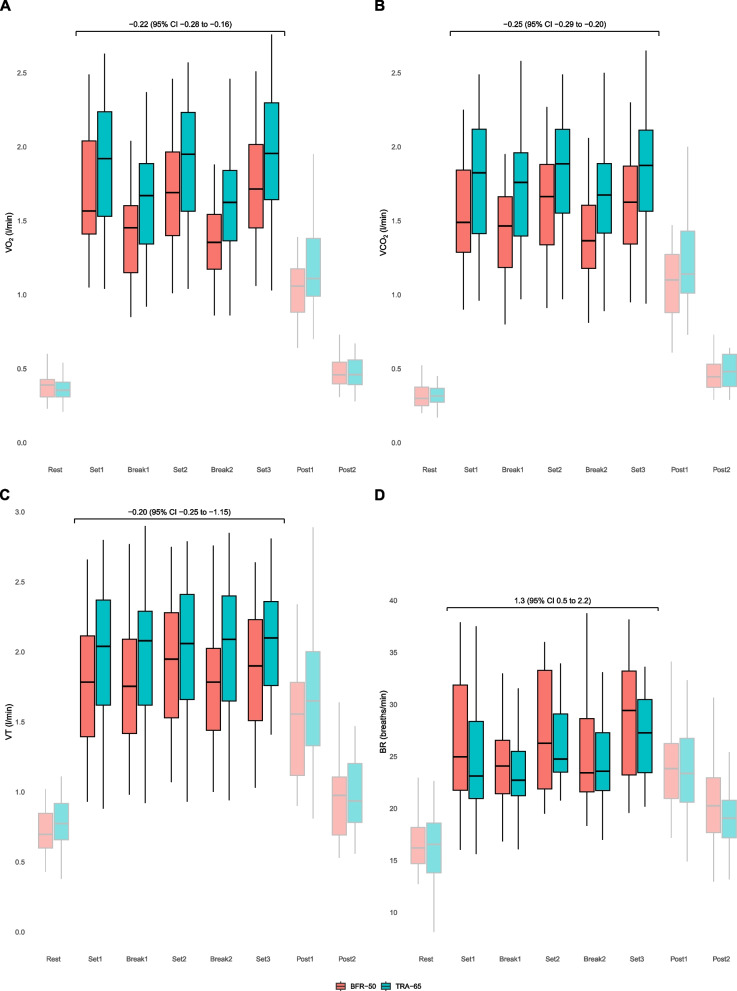
Time course of alterations in oxygen consumption (VO_2_) (**A**), carbon dioxide output (VCO_2_) (**B**), tidal volume (VT) (**C**), and breathing rate (BR) (**D**) during intermittent cycling exercise, represented by BFR-50 (red) and TRA-65 (green). The annotation displays mean difference (95% CI) from linear mixed regression modeling. BFR-50: Blood-flow restriction endurance exercise; TRA-65: Traditional endurance exercise. The box plots display the median (line inside the box), interquartile range (IQR, edges of the box), and whiskers (lines extending from the box). Whiskers represent the range within 1.5 times the IQR from the 25th percentile (Q1—1.5IQR) to the 75th percentile (Q3 + 1.5IQR)

**Fig. 5:**
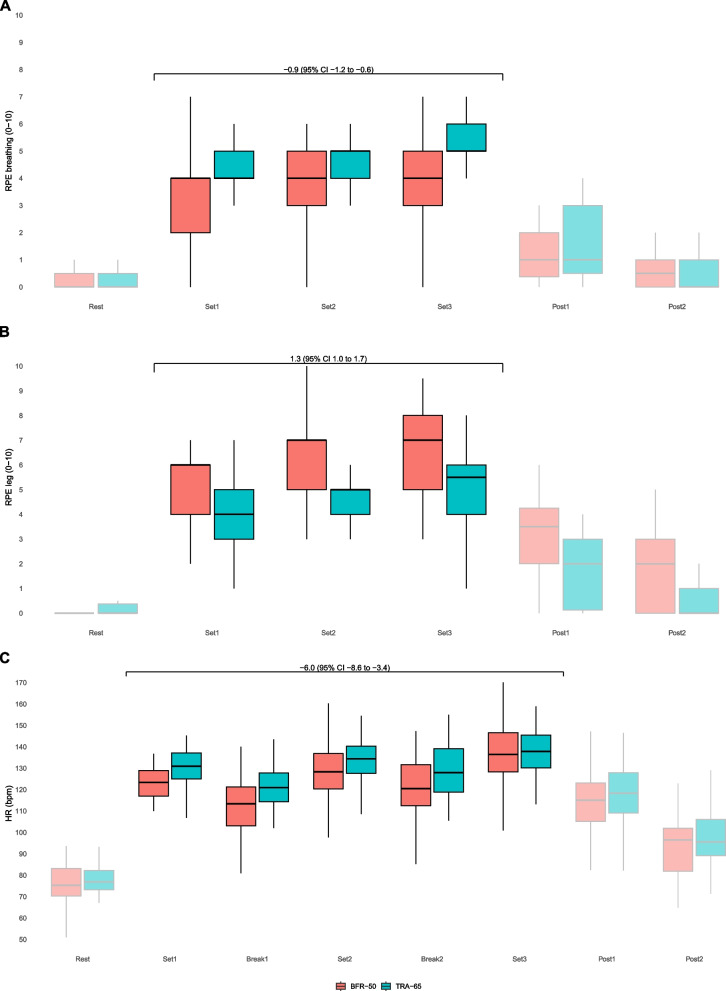
Time course of alterations in the rating of perceived exertion breathing (RPE breathing) (**A**), the rating of perceived exertion leg (RPE leg) (**B**), and heart rate (HR) (**C**) during intermittent cycling exercise as BFR-50 (red) and TRA-65 (green). The annotation displays mean difference (95% CI) from linear mixed regression modeling. BFR-50: Blood-flow restriction endurance exercise; TRA-65: Traditional endurance exercise. The box plots display the median (line inside the box), interquartile range (IQR, edges of the box), and whiskers (lines extending from the box). Whiskers represent the range within 1.5 times the IQR from the 25th percentile (Q1—1.5IQR) to the 75th percentile (Q3 + 1.5IQR)

The cardiorespiratory parameters and RPE data exhibit a characteristic zigzag pattern throughout the interval sets and rest periods, owing to the intermittent nature of the cycling protocol. After exercise, the cardiorespiratory, SpO_2_, and RPE values recovered at Post1 and Post2, as depicted in Figs. 3, 4 and 5, respectively. Supplemental figures S1 (VE), S2 (VCO_2_) and S3 (VO_2_) display the time course of alternation in VE, VCO_2_ and VO_2_ using means out of 10 s intervals throughout the exercise.

Compared with TRA-65, BFR-50 was associated with lower VE (-3.1 l/min [-4.4 to -1.7]), VO_2_ (-2.66 ml/min/kg [-3.44 to -1.87]), VO_2_ (-0.22 l/min [-0.28 to -0.16]), VCO_2_ (-0.25 l/min [-0.29 to -0.20]), VT (-0.20 l [-0.29 to -0.15]), HR (-6.0 bpm [-8.6 to -3.4]), SpO_2_ (-0.6% [-0.8 to -0.4]) and RPE breathing (-0.9 points [-1.2 to -0.6]) (Table 2). Moreover, the RPE of the leg (1.3 points [1.0 to 1.7]) and BR (1.3 breaths/min [0.5 to 2.2]) were higher during BFR-50 than during TRA-65.

**Table 2:** Mixed linear models on mean difference in VE and secondary end-points between traditional endurance exercise (TRA-65) and blood-flow restriction endurance exercise (BFR-50)

	**TRA-65 (*n* = 26)**	**BFR-50 (*n* = 24**)	**Mean differences (*n* = 24)**	**% Differences**	***P*- Value**
**Primary end point**					
VE L/min	51.0 (11.33)	47.8 (11.26)	-3.1 (-4.4 to -1.7)	-6.1 ( -8.62 to -3.38)	< 0.001
**Secondary end points**					
VO_2_ mL/min/kg	24.86 ( 5.97)	22.02 (5.10)	-2.66 (-.3.44 to -1.87)	-12.2 (-8.9 to -15.6)	< 0.001
VO_2_ L/min	1.82 (0.44)	1.59 (0.40)	-0.22 (-0.28 to -0.16)	-14.1 (-16.4 to -11.9)	< 0.001
VCO_2_ L/min	1.78 (0.42)	1.52 (0.35)	-0.25 (-0.29 to -0.20)	-8.7 (-10.7 to -6.8)	< 0.001
VT L	2.07 (0.51)	1.88 (0.51)	-0.20 (-0.25 to -0.15)	3.9 (1 to 6.8)	< 0.001
BR (breaths per min)	25.4 (4.9)	26.6 (6.1)	1.3 (0.5 to 2.2)	-4.5 (-6.6 to -2.5)	0.003
Heart rate (bpm)	130.7 (14.0)	124.7 (17.6)	-6.0 (-8.6 to -3.4)	0.6 (0.4 to 0.8)	< 0.001
SpO_2_ (%)	95.1 (1.2)	95.7 (1.3)	-0.6 (-0.8 to -0.4)	35.3 (27 to 35.3)	< 0.001
RPE leg (0–10)	4.5 (1.6)	5.9. (2.1)	1.3 (1.0 to 1.7)	-19.8 (-34.7 to -4.5)	< 0.001
RPE breathing (0–10)	4.6 (1.4)	- 3.6 (1.5)	-0.9 (-1.2 to -0.6)	-12.2 (-8.9 to -15.6)	< 0.001

Data are presented as means and standard deviations, mean differences with corresponding 95% confidence intervals and percent differences with corresponding 95%. Positive coefficients indicate that traditional exercise gave larger measurements than BFR exercise

*Abbreviations*: *VE* Ventilation, *VO*_*2*_ Oxygen consumption, *VCO*_*2*_ Carbon dioxide output, *VT* Tidal volume, *BR* Breathing rate, *SpO*_*2*_ Peripheral oxygen saturation, *RPE leg* Rating of perceived leg exertion on a scale of 0 to 10 (0 no fatigue; 10 maximum fatigue), *RPE breathing* Ratings of perceived breathing on a scale of 0 to 10 (0 no shortness of breath; 10 maximum shortness of breath)

## Discussion

This is the first study investigating the acute cardiorespiratory response between low-intensity BFR endurance exercise and moderate-intensity traditional endurance exercise in healthy subjects. We observed significantly less VE and RPE breathing during BFR-50 than during TRA-65. Additionally, the RPE of the leg was significantly higher during BFR-50 than during TRA-65.

Our findings indicate a lower level of respiratory work in BFR-50 compared to TRA-65, despite marginal differences in exercise intensity between the two conditions. It appears reasonable to infer that BFR-50 demands less cardiorespiratory effort than does TRA-65, primarily attributable to the reduced intensity associated with BFR-50. However, it is essential to acknowledge that while prior research indicates that the application of BFR during exercise leads to an augmented cardiorespiratory demand in comparison to the same exercise performed without BFR [15–17], our study specifically aimed to compare the cardiorespiratory responses between BFR-50 and TRA-65 exercises. This distinction is crucial because BFR is typically recommended for use at 50% of IPPO, whereas 65% of IPPO is commonly used during rehabilitation in patients with cardiorespiratory limitations [4, 29]. Prior to our study, it was unclear whether BFR-50 elicits a diminished cardiorespiratory response compared to TRA-65 in healthy subjects. Had we observed a higher cardiorespiratory demand during BFR-50, concerns about its suitability in rehabilitation settings in patients with cardiorespiratory limitations would have increased. Our study's findings can be generalized to healthy adults who engage in low- and moderate-intensity endurance exercises, providing valuable insights into the differential cardiorespiratory demands of BFR and traditional endurance exercise protocols.

Cardiorespiratory responses exhibit characteristic kinetics during constant-load exercise [30]. Simply averaging data across intervals may obscure important differences between experimental conditions. Therefore, we analyzed the final 20sec of each phase ("Set1″, "Set2″, "Set3″, "Break1″, "Break2″, "Post1″ and "Post2″) and applied LMM to these data (Table S1). Overall, all respiratory parameters, SpO_2_, RPE, and HR followed similar trends and exhibited values consistent with our presented data in Table 2. Additionally, post hoc pairwise comparisons between conditions and each phase of exercise are provided in Table S2.

We found significantly lower VE, VO_2_, and VCO_2_ with BFR-50 compared to TRA-65. Interestingly, the findings of our study align with prior research examining the disparities between low-intensity BFR endurance exercise and high-intensity traditional endurance exercise [15, 17, 19]. Notably, despite the reduction in the intensity of traditional endurance exercise and increase in the intensity of BFR endurance exercise compared to previous studies, a diminished cardiorespiratory response was observed in BFR-50 as compared to TRA-65. This finding demonstrated that despite the addition of BFR, a 15% absolute difference in exercise intensity still elicits significantly reduced ventilatory effort in healthy subjects. Future research may aim to identify the specific intensity and LOP at which BFR and traditional endurance exercise start to deflect in terms of cardiorespiratory demands.

The lower VE in BFR-50 was accompanied by significantly less RPE breathing compared to TRA-65. This finding is consistent with previous work in healthy individuals investigating BFR endurance training vs. traditional endurance training to task failure [18].The difference in VE between BFR-50 and TRA-65 can be attributed to the smaller VT observed in BFR-50, as both groups exhibited minimal differences in breathing rate (BR) between experimental conditions. Further, group III-IV muscle afferents play a crucial role in exercise-induced sympathoexcitation, hyperpnea, and BR [31]. The accumulation of metabolites during BFR-50 may have led to greater activation of the leg muscles compared to TRA-65 [32]. This could explain the increase in BR but lower VT observed during BFR-50, reflecting a lower mechanical demand compared to TRA-65. However, the increase in BR in BFR-50 compared to TRA-65 is clinically irrelevant.

Overall the interpretation of the magnitude of the difference in VE between BFR-50 and TRA-65 in healthy adults is challenging due to the absence of empirical investigations. However, BFR-50 is interesting for rehabilitation settings, with the aim of making endurance exercise more comfortable. For example, it has been established in individuals with chronic obstructive pulmonary disease (COPD) that a difference of 0.04 (l/min) in peak oxygen uptake is clinically meaningful [33].

In contrast to lower RPE breathing, BFR-50 had a more pronounced effect on the RPE leg than TRA-65. This finding is in line with previous work that emphasized the modulation of the RPE leg in BFR endurance exercise by cuff pressure [15, 17, 19, 34, 35]. Higher cuff pressure results in a greater RPE leg [15, 17, 19, 34, 35]. Generally, the RPE leg is greater during exercise with BFR compared to exercise without BFR. Nevertheless, when comparing low-intensity BFR with high-intensity traditional exercise, the RPE leg appears to be similar or less [17, 19]. Additionally, an increase in the RPE leg is also associated with the application of cuff pressure and the width of the cuff [35].

In our study, we observed a slightly lower HR (mean difference of -6.0 bpm) in BFR-50 compared to TRA-65. Hence, it can be assumed that the potential for endurance performance adaptations may be constrained because the prerequisites for the enhancement of VO_2max_ are an elevated HR and cardiac output during endurance training [36]. Prior studies have provided evidence of higher HR in BFR endurance exercise compared to work-matched traditional endurance exercise [15, 37, 38]. Ozaki et al. [38] reported that adding a standardized LOP of 200 mmHg during low- and moderate-intensity cycling exercise at 20%, 40%, and 60% of VO_2max_ resulted in a 10% increase in HR in healthy people. In our study, additional visits with traditional exercise at 50% IPPO provided information on the specific magnitude of HR increase due to BFR.

Nevertheless, it should be noted that endurance adaptations in BFR training are not solely associated with an elevated HR. BFR endurance training has been shown to induce VO_2max_ augmentation and delays the onset of blood lactate accumulation through metabolic and vascular stimuli [39]. Accordingly, future research should investigate the metabolic and vascular adaptations associated with BFR-50 and TRA-65.

### Limitations

This study has several limitations. First, we matched BFR-50 and TRA-65 by increasing time according to the difference in workload. An alternative approach would have been to account for the total work completed during the intervals, which would have resulted in 2 min 36 s sets for BFR-50. However, pilot testing of our protocol showed that a substantial number of participants would not tolerate BFR while cycling for > 30 s longer and we therefore applied the present approach.

Second, the comparison between TRA-65 and BFR-50 was performed by continuously applying 50% LOP. Cuff pressure might influence acute physiological responses. Therefore, whether similar acute cardiorespiratory responses are observed if either lower or higher cuff pressures or intermittent occlusions are used remains to be investigated. For this study, we derived the intensity and the LOP from best practice guidelines [4]. However, it would be meaningful to determine the minimal LOP at which BFR-50 and TRA-65 start to elicit distinct cardiorespiratory responses. Furthermore, the systolic pressure and mean arterial pressure typically increase during exercise, and the cuff pressure remains at 50% of the initially determined LOP. This may result in less proportional blood flow obstruction during exercise compared to rest [40]. Future studies could aim to quantify the precise reduction during BFR exercise to provide a clearer understanding of its restriction effects during exercise. Third, we did not assess the activity levels or prior BFR training experience of the participants. As a result, we cannot determine if cardiorespiratory responses differ across varying activity levels or if prior experience with BFR impacts these responses. Future studies should consider assessing and reporting these variables to provide a more detailed context for interpreting the findings.

## Conclusion

In healthy subjects, BFR endurance exercise at 50% IPPO and 50% LOP resulted in lower cardiorespiratory work and perceived breathing effort compared to traditional endurance exercise at 65% IPPO. BFR-50 could be an attractive alternative for TRA-65, eliciting less respiratory work and perceived breathing effort while augmenting perceived leg muscle effort.

### Supplementary Information

Additional file 1: Figure S1. Time course of alterations in VE during intermittent cycling exercise, as measured by BFR-50and TRA-65. The line plot was created using means out of 10 s intervals throughout the exercise. VE: minute ventilation; BFR-50: Blood-flow restriction endurance exercise; TRA-65: Traditional endurance exercise

Additional file 2: Figure S2. Time course of alterations in VCO_2_ during intermittent cycling exercise, shown as BFR-50and TRA-65. The line plot was created using means out of 10 s intervals throughout the exercise. VCO_2_: Carbon dioxide output; BFR-50: Blood-flow restriction endurance exercise; TRA-65: Traditional endurance exercise.

Additional file 3: Figure S3.Time course of alterations in VO_2_ during intermittent cycling exercise in the BFR-50and TRA-65. The line plot was created using means out of 10 s intervals throughout the exercise. VO_2_: Oxygen consumption; BFR-50: Blood-flow restriction endurance exercise; TRA-65: Traditional endurance exercise.

Additional file 4

Additional file 5

## Data Availability

The data that support the findings of this study are available from the corresponding author, Dario Kohlbrenner, upon request.

## Abbreviations

BFR: Blood-flow restriction
BFR-50: Low-intensity blood-flow restriction endurance exercise
BR: Breathing rate
CPET: Cardiopulmonary exercise test
HR: Heart rate
IPPO: Incremental peak power output
LOP: Limb occlusion pressure
RPE: Ratings of perceived exertion
RPM: Revolutions per minute
SD: Standard deviation
SpO_2_: Peripheral oxygen saturation
TRA-65: Moderate-intensity traditional endurance exercise
VCO_2_: Carbon dioxide output
VE: Minute Ventilation
VT: Tidal volume
VO_2_: Oxygen consumption
VO_2peak_: Peak oxygen uptake
