# Supplementary figures and images for: Exploring immediate cardiorespiratory responses: low-intensity blood flow restricted cycling vs. moderate-intensity traditional exercise in a randomized crossover trial
Source: BMC Sports Sci Med Rehabil. 2024 Aug 15;16:172. doi: 10.1186/s13102-024-00951-0 (PMC11325739; doi:10.1186/s13102-024-00951-0)

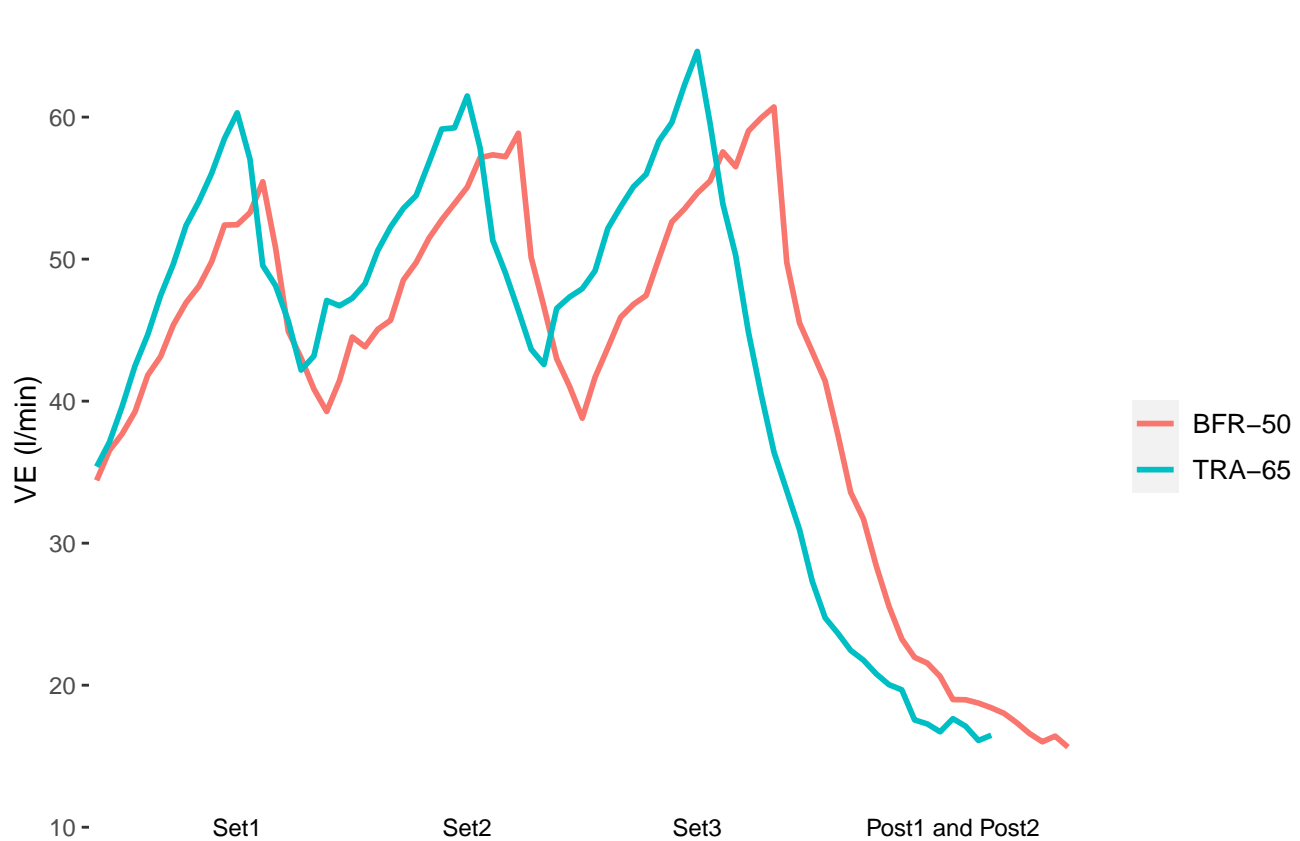

Supplement: Supplementary file 1 — Additional file 1: Figure S1. Time course of alterations in VE during intermittent cycling exercise, as measured by BFR-50and TRA-65. The line plot was created using means out of 10 s intervals throughout the exercise. VE: minute ventilation; BFR-50: Blood-flow restriction endurance exercise; TRA-65: Traditional endurance exercise [file 13102_2024_951_MOESM1_ESM.pdf]

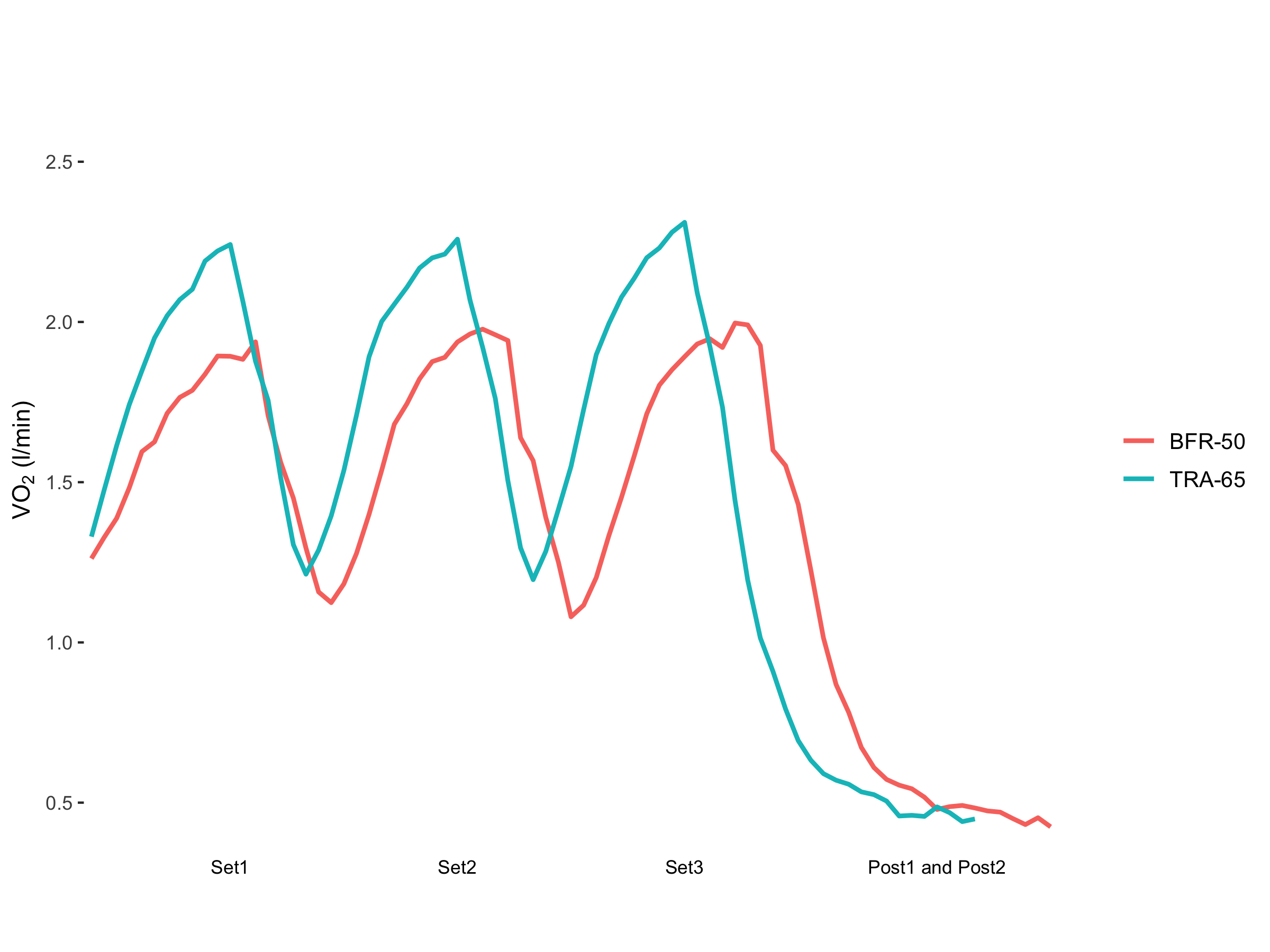

Supplement: Supplementary file 2 — Additional file 2: Figure S2. Time course of alterations in VCO2 during intermittent cycling exercise, shown as BFR-50and TRA-65. The line plot was created using means out of 10 s intervals throughout the exercise. VCO2: Carbon dioxide output; BFR-50: Blood-flow restriction endurance exercise; TRA-65: Traditional endurance exercise. [file 13102_2024_951_MOESM2_ESM.png]

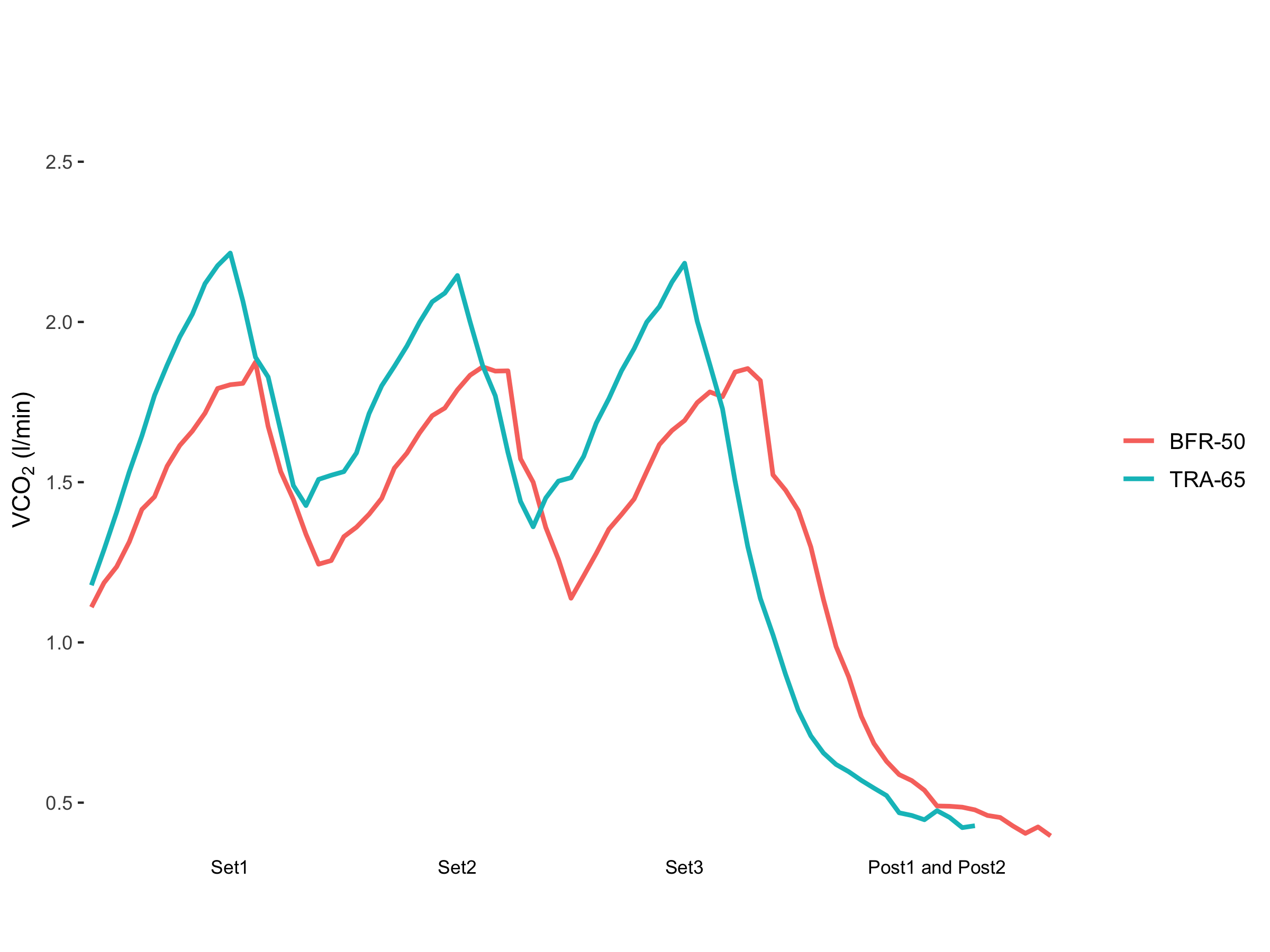

Supplement: Supplementary file 3 — Additional file 3: Figure S3.Time course of alterations in VO2 during intermittent cycling exercise in the BFR-50and TRA-65. The line plot was created using means out of 10 s intervals throughout the exercise. VO2: Oxygen consumption; BFR-50: Blood-flow restriction endurance exercise; TRA-65: Traditional endurance exercise. [file 13102_2024_951_MOESM3_ESM.png]
