# Supplementary material for: Exploring immediate cardiorespiratory responses: low-intensity blood flow restricted cycling vs. moderate-intensity traditional exercise in a randomized crossover trial
Source: BMC Sports Sci Med Rehabil. 2024 Aug 15;16:172. doi: 10.1186/s13102-024-00951-0 (PMC11325739; doi:10.1186/s13102-024-00951-0)
Supplement: Supplementary file 4 — Additional file 4 [file 13102_2024_951_MOESM4_ESM.pdf]

Table S1| Linear mixed models on difference in mean VE and secondary outcomes between traditional and BFR endurance exercise over Set1, Set2, Set3, Break1 and Break 2 (Means final 20sec of each phase)

	TRA-65	BFR-50	Mean Difference	95% CI	% Difference	P Value
VE L/min	54.1 (2.25)	50.7 (2.22)	-3.43	-6.17– -0.68	-6.34%	0.015
VO2 L/min	1.87 (0.08)	1.64 (0.08)	-0.24	-0.37 – -0.11	-12.83%	<0.001
VO2 kg/L/min	25.5 (1.02)	22.7 (1.05)	-2.86	-4.65 – -1.06	-11.22%	0.002
VCO2 L/min	1.88 (0.08)	1.6 (0.08)	-0.28	-0.38 – -0.17	-14.89%	<0.001
VT L	2.12 (0.1)	1.94 (0.1)	-0.18	-0.25 – -0.11	-8.49%	<0.001
BR (breaths per min)	26.2 (1)	26.9 (1)	0.78	-0.20 – 1.75	2.98%	0.120
RER	1.03 (0.01)	1 (0.01)	-0.03	-0.05 – -0.01	-2.91%	0.017
Heart rate (bpm)	132 (2.8)	126 (2.9)	-6	-10.9– -1.1	-4.55%	0.016
SpO2 (%)	95.04 (0.22)	95.65 (0.22)	0.61	0.33 – 0.89	0.64%	<0.001
RPE leg (0-10)	4.5 (0.3)	6 (0.3)	1.6	1.2 – 1.9	26.67%	<0.001
RPE breathing (0-10)	4.5 (0.3)	3.5 (0.3)	-1	-1.3 – -0.7	-22.22%	<0.001

NOTE. Data are presented as means and standard deviations, mean differences with corresponding 95% confidence intervals and percent differences with corresponding 95%. Positive coefficients indicate that traditional exercise gave larger measurements than BFR exercise.

Abbreviations: VE: Ventilation; VO_2: Oxygen consumption; VCO_2: Carbon dioxide output; VT: Tidal volume; BR: Breathing rate; RER: Respiratory exchange ratio SpO_2: Peripheral oxygen saturation; RPE leg: Rating of perceived leg exertion on a scale of 0 to 10 (0 no fatigue; 10 maximum fatigue); RPE dyspnea: Ratings of perceived dyspnea on a scale of 0 to 10 (0 no shortness of breath 10 maximum shortness of breath)
