# Supplementary material for: Exploring immediate cardiorespiratory responses: low-intensity blood flow restricted cycling vs. moderate-intensity traditional exercise in a randomized crossover trial
Source: BMC Sports Sci Med Rehabil. 2024 Aug 15;16:172. doi: 10.1186/s13102-024-00951-0 (PMC11325739; doi:10.1186/s13102-024-00951-0)
Supplement: Supplementary file 5 — Additional file 5 [file 13102_2024_951_MOESM5_ESM.pdf]

Supplement Table 2 | Post-hoc linear mixed models on difference in mean VE and secondary outcomes between traditional and BFR endurance exercise in the individual exercise phases (Means final 20sec of each phase)					
	**TRA-65**	**BFR-50**	**Mean Difference**	**95% CI**	**% Difference**
**Set1**					
VE L/min	59.7 (2.33)	54.9 2.35	-4.86	-7.36 to -2.35	-8.14%
VO_2 L/min	2.25 (0.09)	1.93 (0.09)	-0.31	-0.36 to -0.27	-13.78%
VO_2 mL/kg/min	30.6 (1.15)	26.8 (1.16)	-3.80	-4.82 to -2.78	-12.42%
VCO_2 L/min	2.21 (0.09)	1.86 (0.09)	-0.35	-0.41 to -0.28	-15.84%
VT L	2.28 (0.11)	2.12 (0.12)	-0.16	-0.26 to -0.05	-7.02%
BR (breaths per min)	27.2 (1.2)	27.1 (1.22)	-0.06	-1.68 to 1.56	-0.22%
RER	0.984 (0)	0.97 (0.01)	-0.02	-0.04 to 0.00	-2.03%
Heart rate (bpm)	141 (2.6)	132 (2.6)	-9	-12.2 to -5.7	-6.38%
SpO_2 (%)	95.2 (0.38)	95.4 (0.4)	0.13	-0.90 to 1.16	0.14%
RPE leg (0-10)	4 (0.3)	5.2 (0.3)	1.2	0.6 to 1.7	30.00%
RPE breathing (0-10)	4.1 (0.2)	3.1 (0.2)	-1	-1.5 to -0.5	-24.39%
**Set2**					
VE L/min	60.6 (2.43)	58.2 (2.45)	-2.43	-4.81 to -0.05	-4.01%
VO_2 L/min	2.24 (0.09)	1.98 (0.09)	-0.27	-0.33 to -0.21	-11.61%
VO_2 mL/kg/min	30.5 (1.17)	27.4 (1.18)	-3.16	-4.31 to -2.01	-10.36%
VCO_2 L/min	2.12 (0.09)	1.87 (0.09)	-0.26	-0.33 to -0.19	-12.26%
VT L	2.24 (0.11)	2.11 (0.11)	-0.14	-0.23 to -0.04	-6.25%
BR (breaths per min)	27.8 (1.07)	28.6 (1.1)	0.80	-1.19 to 2.79	2.88%
RER	0.95 (0.01)	0.95 (0.01)	-0.00	-0.02 to 0.01	-0.00%
Heart rate (bpm)	145 (3.2)	137 (3.4)	-8	-15. to -1.00	-5.52%
SpO_2 (%)	94.8 (0.28)	95.7 (0.29)	0.98	0.34 to 1.61	1.03%
RPE leg (0-10)	4.4 (0.3)	6.3 (0.3)	1.8	1.2 to 2.5	40.91%
RPE breathing (0-10)	4.5 (0.3)	3.6 (0.3)	-0.9	-1.4 to -0.4	-20.00%
**Set3**					
VE L/min	63.5 (2.67)	60.3 2.70	-3.21	-5.98 to -0.44	-5.06%
VO_2 L/min	2.31 (0.09)	2.01 (0.09)	-0.30	-0.35 to -0.26	-12.99%
VO_2 mL/kg/min	31.4 (1.14)	27.7 (1.15)	-3.67	-4.71 to -2.63	-11.69%
VCO_2 L/min	2.16 (0.09)	1.87 (0.09)	-0.30	-0.36 to -0.23	-13.89%
VT L	2.24 (0.11)	2.10 (0.11)	-0.14	-0.26 to -0.03	-6.25%
BR (breaths per min)	29.2 (1.18)	29.7 (1.21)	0.49	-1.65 to 2.63	1.68%
RER	0.94 (0.01)	0.93 (0.01)	-0.01	-0.02 to 0.01	-1.06%
Heart rate (bpm)	147 (3.5)	145 (3.7)	-2.1	-10.3 to 6.1	-1.43%
SpO_2 (%)	94.7 (0.24)	95.71 (0.25)	0.97	0.43 to 1.51	1.02%
RPE leg (0-10)	4.9 (0.3)	6.7 (0.3)	1.8	1.0 to 2.5	36.73%
RPE breathing (0-10)	5 (0.3)	3.9 (0.3)	-1.1	-1.7 to -0.4	-22.00%
**Break1**					
VE L/min	43.0 (2.02)	40.3 (2.07)	-2.69	-6.28 to 0.89	-6.26%
VO_2 L/min	1.29 (0.07)	1.16 (0.07)	-0.13	-0.27 to 0.01	-10.08%
VO_2 mL/kg/min	17.6 (0.94)	16.1 (1)	-1.46	-3.37 to 0.46	-8.30%
VCO_2 L/min	1.48 (0.07)	1.26 (0.07)	-0.22	-0.36 to -0.08	-14.86%
VT L	1.93 (0.1)	1.71 (0.1)	-0.21	-0.34 to -0.09	-10.88%
BR (breaths per min)	23.1 (0.94)	24.3 (0.97)	1.18	-0.81 to 3.17	5.11%
RER	1.16 (0.02)	1.09 (0.02)	-0.06	-0.10 to -0.03	-5.17%
Heart rate (bpm)	110 (3.3)	104 (3.4)	-5.9	-11.7 to -0.1	-5.36%
SpO_2 (%)	95.4 (0.26)	96 (0.26)	0.53	0.08 to 0.98	0.56%
**Break2**					
VE L/min	43.3 (2.27)	39.9 (2.32)	-3.36	-6.96 to 0.24	-7.75%
VO_2 L/min	1.27 (0.07)	1.12 (0.07)	-0.15	-0.27 to -0.04	-11.81%
VO_2 mL/kg/min	17.4 (1)	15.7 (1)	-1.73	-3.37 to -0.08	-9.89%
VCO_2 L/min	1.41 (0.07)	1.18 (0.07)	-0.24	-0.37 to -0.11	-16.96%
VT L	1.91 (0.1)	1.65 (0.1)	-0.26	-0.36 to -0.16	-15.76%
BR (breaths per min)	23.3 (1.1)	25.1 (1.13)	1.81	-0.19 to 3.80	7.78%
RER	1.11 (0.02)	1.05 (0.02)	-0.06	-0.09 to -0.04	-5.41%
Heart rate (bpm)	116 (3.2)	112 (3.3)	-4	-9.9 to 2.0	-3.57%
SpO_2 (%)	95.1 (0.26)	95.4 (0.27)	0.30	-0.35 to 0.95	0.32%
**Post1**					
VE L/min	22.9 (1.44)	22 (1.46)	-0.9	-2.89 to -1.1	-3.96%
VO_2 L/min	0.58 (0.04)	0.56 (0.04)	-0.02	-0.07 to -0.02	-3.45%
VO_2 mL/kg/min	7.8 (0.38)	7.6 (0.39)	-0.12	-0.7 to 0.47	-1.54%
VCO_2 L/min	0.63 (0.04)	0.59 (0.04)	-0.04	-0.09 to -0.00	-7.69%
VT L	1.15 (0.76)	1.1 (0.76)	-0.04	-0.13 to -0.04	-3.48%
BR (breaths per min)	20.8 (0.85)	20.7 (0.88)	-0.09	-1.85 to 1.67	-0.43%
RER	1.1 (0.02)	1.06 (0.02)	-0.04	-0.06 to -0.02	-3.64%
Heart rate (bpm)	99 (3.1)	99 (3.2)	0.02	-6.1 to 6.1	0.02%
SpO_2 (%)	95.63 (0.21)	95.36 (0.21)	-0.26	-0.7 to 0.18	-0.27%

RPE leg (0-10)	1.6 (0.31)	3.2 (0.32)	1.6	0.9 to 2.2	50%
RPE breathing (0-10)	1.5 (0.24)	1.3 (0.24)	-0.3	-0.7 to 0.2	-20%
**Post2**					
VE L/min	16.2 (0.87)	16 (0.88)	-0.24	-1.78 to -1.3	-3.64%
VO_2 L/min	0.44 (0.02)	0.44 (0.02)	-0.01	-0.04 to -0.03	-0.02%
VO_2 mL/kg/min	6.1 (0.26)	6.01 (0.26)	0.02	-0.47 to 0.51	-0.27%
VCO_2 L/min	0.42 (0.02)	0.4 (0.02)	-0.02	-0.05 to 0.02	-1.48%
VT L	0.89 (0.56)	0.9 (0.57)	0.01	-0.09 to 0.11	-2.27%
BR (breaths per min)	18.9 (0.92)	18.7 (0.94)	-0.18	-.2.51 to 2.15	-1.52%
RER	0.95 (0.01)	0.93 (0.01)	-0.02	-0.05 to 0.01	-0.58%
Heart rate (bpm)	92 (2.6)	90 (2.8)	-2.3	-7.0 to 2.5	-2.53%
SpO_2 (%)	95.34 (0.2)	94.9 (0.21)	-0.43	-0.79 to 0.08	-0.45%
RPE leg (0-10)	0.7 (0.25)	1.7(0.26)	1	0.45 to 1.59	58.82%
RPE breathing (0-10)	0.6 (0.18)	0.7 (0.17)	0.2	-0.1 to 0.5	14.29%

NOTE. Data are presented as means and standard deviations, mean differences, and corresponding 95% confidence intervals.

Abbreviations: VE: Ventilation; VO_2: Oxygen consumption; VCO_2: Carbon dioxide output; VT: Tidal volume; BR: Breathing rate; RER: Respiratory exchange ratio SpO_2: Peripheral oxygen saturation; RPE leg: Rating of perceived leg exertion on a scale of 0 to 10 (0 no fatigue; 10 maximum fatigue); RPE dbreathing: Ratings of perceived breathing on a scale of 0 to 10 (0 no shortness of breath 10 maximum shortness of breath)
